# Supplementary material for: Content-rich biological network constructed by mining PubMed abstracts
Source: BMC Bioinformatics. 2004 Oct 8;5:147. doi: 10.1186/1471-2105-5-147 (PMC528731; doi:10.1186/1471-2105-5-147)
Supplement: Additional File 2 — The original results of the above study (non-essential files are deleted to keep the file size under the limit set by BMC bioinformatics). [file 1471-2105-5-147-S2.bz2 › chilibotAdditionalFile2/dip20/276ID1827203E1096/html/ATF4_JUN.html]

 


 **ATF4** and **JUN** 
  
Found 16 abstracts in PubMed, retrieved 16.  
 

 What does Google say? 
 PDF only 
| .edu only 

---

**Interactive relationship** (e.g. stimulation, inhibition, etc)

**Inhibitory relationship**- In contrast, cotransfection with the  **ATF4**  expression plasmid suppressed the promoter activation mediated by ATF2 and  **Jun**  family members.  Ref: 9511728 Exp Cell Res, 1998
**Stimulatory relationship**- In order to determine the identities of individual B ZIP proteins in various AP 1  [ **JUN** ]  complexes we tested the effect of dominant negative mutants to the B ZIP proteins c Fos, ATF2,  **ATF4**  and CCAAT enhancer binding protein C EBP on the activities of the collagenase and c  **Jun**  promoters.  Ref: 11736649 Biochem J, 2001
**Neutral relationship**- In contrast, a dominant negative CREB2  [ **ATF4** ]  mutant did not impair ATF2 mediated transcriptional activation, suggesting that CREB2  [ **ATF4** ]  exhibits a different dimerization specificity than ATF2 or c  **Jun** .  Ref: 12751796 Biol Chem, 2003
- In addition to its own family members,  **ATF4**  can also form heterodimers with other related but distinct bZIP proteins such as the C EBP, AP 1  [ **JUN** ]  and Maf families, which may give rise to a variety of combinatorial diversity in gene regulation.  Ref: 10096021 Genes Cells, 1998

**Non-interactive relationship** (e.g. studied together, co-existance, homology, etc.)

- Electrophoretic mobility shift EMS analyses demonstrate that the LP1 CRE like elements interact with distinct subsets of neuronal ATF CREB and  **Jun**  Fos proteins including CREB 1, CREB 2  [ **ATF4** ] , ATF 1, and JunD.  Ref: 9845850 J Biomed Sci< MedlineTA>J Biomed Sci, 1998
- Activation of the rat cyclin A promoter by ATF2 and  **Jun**  family members and its suppression by  **ATF4** .  Ref: 9511728 Exp Cell Res, 1998
- Binding of CREB 2  [ **ATF4** ] , ATF 2, USF 2, and c  **Jun**  transactivators to a wild type and CRE mutated oligonucleotide was analyzed by a novel DNA binding assay.  Ref: 12057991 Circulation, 2002
- This study demonstrates that the location of the gene for murine ATF 4  [ **ATF4** ]  is not linked to the genes for  **JUN**  family members, CREB1 and CREB2  [ **ATF4** ] .  Ref: 8432535 Genomics, 1993
- Within the nucleus, Nrf1 and Nrf2 are recruited to the ARE as heterodimers with either small Maf proteins, FosB, c  **Jun** , JunD, activating transcription factor 2 ATF2 or  **ATF4** .  Ref: 11689285 Cancer Lett, 2001
- These genes included genes involved in cell cycle regulation GADD45, CIP1 WAF1, in stress response HSP70, HSP40, HSP86, in apoptosis GADD153, tristetraproline and genes encoding transcription factors EGR 1, ETR 101, c  **JUN** ,  **ATF4** .  Ref: 11750059 Biochim Biophys Acta, 2001
- Antibody supershift EMSA and Western blot analysis confirmed that increased binding of these factors correlated with LPS induced increases in nuclear concentrations of AP 1  [ **JUN** ]  c  **Jun** , phosphorylated c  **Jun** ,  **Jun**  D, and  **Jun**  B, C EBPbeta, NF kappaB p50, p65, and c Rel, CREB CREB 1, CREB 2  [ **ATF4** ] , and ATF 2, and AP 2alpha proteins.  Ref: 12662898 Toxicol Appl Pharmacol, 2003
- We investigated by RT PCR whether PROG activated expression of transcription factors Egr 1 Egr 2, Egr 3, c  **jun** ,  **jun**  B,  **jun**  D, c Fos, Fos B, Fra 1, Fra 2, CREB, ATF 4  [ **ATF4** ] , SCIP and Sox 10 in cultured Schwann cells.  Ref: 11750070 Brain Res Mol Brain Res, 2001
- Tax was demonstrated to interact directly with CREB1 but not with other bZIP proteins, including CREB2  [ **ATF4** ]  and  **Jun** .  Ref: 7745688 J Virol, 1995
- In contrast, expression of c  **jun**  and activating transcription factor 4  [ **ATF4** ]  mRNAs remained constant.  Ref: 10915218 J Mol Endocrinol, 2000
